# 

**Published:** 1988-08

**Authors:** 


Copyright © Clinical Press Ltd 1988.

Published quarterly and distributed free to all doctors in the West of England. Overseas subscription £12.

### Contents

34 **Domestic Medicines. An irreverent Look at Health Education**
Martin Crossfil

38 **Simple Audit of Trainer/Trainee consultation profiles as a method of assessing whether non-structured surgeries give adequate training**
F. D. Skerrett, Michael Black, Regan Wooding, Ciarnon Kelly.

40 **Aneurysm of the interatrial septum and mitral valve proplapse—an aetiological association?**
K. S. Channer, R. Bolton, J. Russell Rees, P. Wilde.

44 **Sweet's Syndrome and Erythema Nodosum**
C. E. H. Grattan, C. T. C. Kennedy, S. C. Glover, R. J. Mann

46 **From our Correspondents—'Concrete Cancer at the Royal Devon and Exeter Hospital, The Lithotriptor at Southmead Hospital, Motors medicine and Gilbert Lang.**

48 **From our Foreign Correspndents—Surgical Safari, Anastomosis workshop in Saudi Arabia.**

50 **Obituary—Dr Dennis Wright.**
**Book Reviews—General Pathology—Walters and Israel,**
**Pocket Endocrinology—Martin Hartog.**

51 **South West Surgical Club—Meeting in Truro and Penzance.**

